# Relationships between Self-Efficacy, Job Instability, Decent Work, and Life Satisfaction in A Sample of Italian, Swiss, and Spanish Students

**DOI:** 10.3390/ejihpe13020023

**Published:** 2023-01-28

**Authors:** Andrea Zammitti, Celia Moreno-Morilla, Soledad Romero-Rodríguez, Paola Magnano, Jenny Marcionetti

**Affiliations:** 1 Department of Educational Sciences, University of Catania, 95124 Catania, Italy; 2 Department of Research and Assessment Methods in Education, School of Education, University of Seville, Campus Pirotecnica s/n, 41013 Seville, Spain; 3 Faculty of Human and Social Sciences, Kore University, Cittadella Universitaria, 94100 Enna, Italy; 4 Department of Education and Learning, University of Applied Sciences and Arts of Southern Switzerland, 6600 Locarno, Switzerland

**Keywords:** job instability, decent work, self-efficacy, life satisfaction, career

## Abstract

Recent research has shown that self-efficacy has a positive relationship with life satisfaction and with the perception of access to decent work. On the other hand, a perception of instability regarding the profession is negatively correlated with these dimensions. Few authors have studied these constructs within the same research. Therefore, the aim of the study was to fill this gap in the literature by testing a structural equation model in which the perception of access to decent work could mediate between perceived self-efficacy in one’s training and life satisfaction, and between perceived instability of the profession and life satisfaction. Data was collected through an online research survey. Five hundred and seventeen university students (104 males and 413 females) aged between 18 and 30 years (M = 22.50; ds = 2.61) from three different countries participated: 181 were Italian, 173 were Swiss, and 163 were Spanish. The results only partially confirmed our model. The idea of finding a decent work mediates the relationship between perceived job instability and life satisfaction, but not between self-efficacy and life satisfaction. Perceived self-efficacy together with the idea of finding a decent work have a direct effect on life satisfaction. In career development, counselors must take into account what the perception of job instability entails for students, which may be demotivating and not allow future workers to imagine a decent job.

## 1. Introduction

The features of the so-called risk society [1,2] have been emphasized during the last two years, due to the pandemic, which has exacerbated the uncertainty, instability, and feeling of insecurity that characterize the socioeconomic and labor context in the third millennium. As underlined very recently by Nemteanu, Dinu, and Dabija [3], changes in organizational dynamics have occurred. Many organizations have considerably decreased their activity [4], reducing jobs or employee working hours, leading to lower productivity and organizational competitiveness [5]. Previous studies have shown that job instability and job insecurity are related to low levels of job satisfaction [6], subjective well-being [7,8], and life satisfaction [9]. This is even more true for young people; we agree with Nunn and colleagues [10] that young people’s lives were already precarious before the pandemic (e.g., [11,12]), as “precarity is the condition of our time” [13] (p. 20), primarily in relation to the labor market. In fact, the pandemic has only additionally amplified the existing challenges in youth transition toward the labor market. Young people are significantly more likely than older adults to have experienced job loss and to be unemployed; young people, already before the pandemic, had to face higher under/unemployment rates [10]. They are also more likely to be precarious workers without access to formal entitlements and protections [11]. Using Nunn and colleagues’ words [10] (p. 432) “the pandemic has eroded young people’s confidence about future work and careers and increased uncertainty among those undertaking key transitions ([14,15,16])”.

Accordingly, adopting the Conservation of Resources theory (COR; [17]), individuals who have access to and can draw upon a wider source of resources would better cope with the stress and uncertainty of job insecurity; among the positive personality strengths, that can significantly impact on managing the perception of job instability, the self-efficacy is identified as an important personal resource. Self-efficacy is the core concept of Bandura’s social cognitive theory [18]; it is defined as the beliefs about one’s ability to behave effectively to achieve a goal or perform a task [19]. The role of self-efficacy beliefs in the academic context has been widely deepened, showing that self-efficacy beliefs are significant predictors of academic achievement, continuance, performance, and persistence [20,21,22,23,24]. Consistently with the framework of Social-Cognitive Career Theory (SCCT), self-efficacy predicts academic satisfaction [25], which in turn, affects overall life satisfaction. According to COR, high self-efficacy may lessen the impact of the negative effect of job instability on life satisfaction, since individuals high in self-efficacy may perceive that they have the capacity to successfully cope with the potential difficulties to access the labor market or obtain decent work [26]. Following Lau and Knardahl’s reasoning [27], job instability could be an antecedent of life dissatisfaction and this effect could be stronger among individuals who reported low self-efficacy.

Imagining positive or negative scenarios characterized by a satisfactory or unsatisfactory job can affect the behavioral intention and the subsequent actions [28] that individuals will implement in active research of a job. This dynamic will lead the students in a university-to-work transition to be proactive or passive and discouraged from the current fluid situation of the job market [29]. The university students’ expectations on work are core aspects of career education and vocational guidance process. In a previous study, Zammitti and colleagues [30] found that students who have a more complex concept of work also show greater conviction in the possibility of being able to reach their goals and in the ability to adapt to changes in the world of work. The perception that people have about work is important as it can greatly influence the way individuals make choices and decisions, characterizing their career and life [31].

Stating these premises, the purpose of the present study is to explore the relationships between student self-efficacy, perceived job instability, perception of access to decent work, and life satisfaction in three groups of Italian, Swiss, and Spanish university students.

### Perception of Access to Decent Work

The psychological concept of work [32] includes several core meanings: the expression of individuals’ identity, competencies, and interests; the possibility to gain social status, prestige, and power; and the opportunity to participate in social exchange and providing something economically or socially meaningful to the community. However, the work is not often decent work. Today’s society is characterized by poor growth and development prospects [33] and labor market turbulence increases job instability [34]; precariousness, instability, and insecurity have, as effect, an increase in unemployment which, in turn, leads to the spread of illegal, low-wage, unsafe working conditions. In a word, indecent work is very common among young people at the beginning of their careers [35], causing stress and anxiety [36].

The perception of access to decent work refers to the perception of the opportunity to obtain productive and satisfactory work in conditions of freedom, equity, security, respect for human rights, prospects for individual development, and social integration [37]. There is a growing body of literature on decent work published in the last years. According to the literature review conducted by Haiming and Yan [38], the existent studies on decent work can be organized into three perspectives: security, equity, and self-value. The perspective of safety and security implies the respect of laws and rules; the perspective of equity is referred to the protection of the rights and the dignity of workers. The shift toward the perspective of self-value has been a favorite by a renewed interest toward humanism in organizations. This highlights the importance of the wellbeing of the workers through the re-appropriation of the meaningfulness and the values of work, which are antecedents of satisfaction in various life domains. Finally, empirical studies have found a positive effect of decent work on life satisfaction in different European samples [39,40,41,42].

## 2. Method

### 2.1. Procedure and Participants

Using G*Power version 3.1.9.7 [43,44], an a priori power analysis was conducted. This software allows you to calculate the sample size necessary to detect an effect of a given size. The computation of the right sample size has become a very important aspect in research [45,46] because samples that are too small or too large can produce biased results [47]. First, we calculated the minimum sample size necessary to estimate the differences between three groups by means of an ANOVA analysis. The parameters indicated in the literature were: a medium effect size of 0.25 (Effect size f^2^ = 0.25) with α = 0.05, minimum Power (1 − β) = 0.95 and Number of groups = 3. The analysis indicated a number of 252 participants as the minimum sample size. Second, we calculated the minimum sample size to predict life satisfaction with three predictor variables (student self-efficacy, perceived job instability and access to decent work). The parameters indicated were a medium effect size of 0.15 (Effect size f^2^ = 0.15) with α = 0.05, minimum Power (1 − β) = 0.95 and Number of predictors = 3. In this case, a minimum sample of 119 participants was necessary.

After conducting these analyses, a research protocol specifying the voluntary participation of the students was created. Convenience sampling was used in this study. Participants were recruited during class hours at their university. The students were asked to fill out a research protocol in which the details of the study were indicated: those responsible for the research, information on the anonymous and aggregate processing of the data, and the absence of risks to personal well-being. In this way, all the principles laid down in the code of ethics of the Italian Association of Psychology [48] were respected.

To participate in this research, the following inclusion criteria were considered: minimum age of 18 years and maximum of 30 years, possessing student status and residing in one of the indicated territories (Sicily, Canton Ticino or Andalusia). To verify that participants possessed these characteristics, the protocol included initial questions on these aspects. The protocol was administered in Italian in Sicily and Canton Ticino, as the official language in these areas is Italian. For participants of Spanish nationality, the protocol was translated into Spanish by a bilingual expert who did not belong to the research team (except for the validated measures of which the existing Italian or Spanish version was used).

The three regions where the data were collected are characterized by similarities and differences. All regions are southern in relation to the other regions of the country in which they are located. Andalusia and Sicily are characterized by high unemployment rates. In fact, in 2021, Eurostat highlighted that among the regions of Europe with the highest levels of unemployment there were Andalusia (21.7%; 35.5% youth unemployment rate) and Sicily (18.7%; 40.1% youth unemployment rate), while in Canton Ticino there was a much lower percentage of unemployment (7.9%; 12.1% youth unemployment rate) (Source: https://ec.europa.eu). However, also in the Canton of Ticino, the unemployment rate was higher than the Swiss national average (5.1%) (https://ec.europa.eu).

The initial sample consisted of 558 participants. Ten questionnaires were eliminated because they were incomplete and 31 because they did not meet the age requirement of participants being over 30 years old. Hence, the final sample consisted of 517 participants, of whom 104 (20.1%) were males and 413 (79.9%) females, aged between 18 and 30 years (M = 22.50; ds = 2.61). All participants were students and not working at the time the protocol was completed. The nationality of the participants was as follows: 181 were Italian, 173 were Swiss, and 163 were Spanish. The characteristics of the sample are summarized in Table 1. Based on the a priori analysis previously conducted, our sample was appropriate to conduct an ANOVA analysis and to test the hypothesized model.

### 2.2. Measures

The research protocol included the following sections:

#### 2.2.1. Biographical Data

Participants indicated their age, gender, and their status (student or worker).

#### 2.2.2. Measurement of Student Self-Efficacy

To evaluate student self-efficacy, we used five items adapted from the Patterns of Adaptive Learning Scales (PALS; [49]). The items were translated in Italian and Spanish and adapted for this study. An item example is “I’m certain I can master the skills taught in this training”. Participants are asked to indicate how well each statement fits themselves on a six-point Likert scale, from 1 = not true at all, to 6 = completely true. Cronbach’s alpha coefficient in this study was 0.91.

#### 2.2.3. Measurement of Perceived Job Instability

To evaluate perceived job instability, four items adapted from De Witte et al. [50] and translated in Italian and Spanish were used. An item example is “I think the profession in which I am training will soon change for the worse”. The participant was required to indicate the degree of agreement/disagreement on a five-point Likert scale (from 1 = I totally disagree, to 5 = I totally agree). Cronbach’s alpha coefficient in this study was 0.83.

#### 2.2.4. Measurement of Access to Decent Work

To evaluate student’s perception of access to decent work we used the following statement: “Decent work is an employment that meets the minimum acceptable standards for a good life. Do you think it is easy for you, in your region, to find decent work today?”. Participants were asked to indicate the answer on a six-point Likert scale from 1 “not at all easy” to 6 “very easy”.

#### 2.2.5. Measurement of Life Satisfaction

Life Satisfaction was evaluated with the Satisfaction With Life Scale (SWLS; [51]) using both the Italian [52] and Spanish [53] version. This measure is composed of five items. An item example is “If I could live my life over, I would change almost nothing”. Participants had to indicate their degree of agreement or disagreement with each statement. They responded to each item on a seven-point Likert scale from 1 = I totally disagree, to 7 = I totally agree. Cronbach’s alpha coefficient in this study was 0.88.

### 2.3. Data Analysis

SPSS software was used for preliminary analyses (descriptive statistics, differences between groups and correlations). AMOS software [54] was used to test the hypothesized structural equation modeling (SEM) model. Before testing this model, properties of the measurement model were assessed. To do this, a confirmatory factor analysis (CFA) to determine to what extent the variance of the common method was an issue was performed according to Harman’s single factor test. This allowed us to compare the hypothesized model with a single-factor model. Within the latter, all items were loaded on the same, single factor. As suggested by the literature, the indices used to assess whether a model is good or not are as follows: a χ^2^ ratio per degrees of freedom (χ^2^/df) of less than 3, Tucker-Lewis index (TLI) and comparative fit index (CFI) which must have a value greater than 0.90 [55], root mean square error of approximation (RMSEA) which must be less than 0.08 [56], and standardized root mean square residual (SRMR) which must be less than 0.08 [57]. To compare the two models, the Akaike Information Criterion (AIC) was used. In this case, the interpretation is that the lower value indicates a better model fit [58,59]. Then, 95% confidence intervals for direct, simple indirect and total indirect mediation effects were estimated using bootstrapping on 5000 samples.

Finally, with the aim of investigating whether the estimated effects were similar for the three different groups, invariance was assessed by examining the changes in fit in the configural, weak invariance and structural models. It is recommended in the literature that the assumption of invariance between models is considered acceptable if ΔCFI < .01 and ΔRMSEA < 0.015 [60].

## 3. Results

### 3.1. Preliminary Analyses

In the first phase, we checked whether there were any differences in the three groups of students considered, about gender and age (to view the descriptive statistics by gender and age, consult Table 1). No statistically significant differences emerged for gender (χ^2^_(1)_ = 2.33, *p* = 0.31) and age (*F*_(2, 514)_ = 0.10, *p* = 0.90). We then assessed if there were differences between the three national groups on the constructs analyzed. The analyses showed that only the dimension of student self-efficacy did not show statistically significant differences. Spanish students had higher levels of perceived job instability and life satisfaction than Italian and Swiss students. Swiss students believe they can access decent work more easily than Italian and Spanish students. The results of this analysis are summarized in Table 2.

We then calculated the correlations between the investigated constructs and with age. The results showed that age correlated positively with student self-efficacy. Perceived job instability correlated negatively with student self-efficacy, access to decent work, and life satisfaction. Student self-efficacy correlated positively with life satisfaction. Finally, access to decent work correlated positively with life satisfaction. The results of this analysis are summarized in Table 3.

### 3.2. CFA of the Measures

Since all variables considered were measured from the same source, a bias in the common method may have occurred. To test this, we conducted a CFA according to Harman’s single factor test. By comparing the hypothesised model and a single-factor model (in which all items are loaded on one factor), we verified that the former model provided a better fit (hypothesized model: χ^2^_(85)_ = 3.13, *p* < 0.00, CFI = 0.96, SRMR = 0.04, RMSEA = 0.64, and AIC = 335.91; 1-factor model: χ^2^_(90)_ = 24.95, *p* < 0.00, CFI = 0.49, SRMR = 0.19, RMSEA = 0.22, and AIC = 2305.35). These differences were found to be statistically significant when comparing the χ^2^ values and degrees of freedom of the models: Δχ^2^(5) = 21.82 (*p* < 0.001). These results show that there was no evidence of common method bias in the data.

### 3.3. Structural Equation Model

To test our hypotheses, we applied structural equation model analysis. In the model access to decent work was set as a partial mediator of the student self-efficacy-life satisfaction and perceived job instability-life satisfaction relationships. The main fit indices suggested that the model fit the data adequately: χ^2^_(85)_ = 3.13, p < 0.00, TLI = 0.94, CFI = 0.96, SRMR = 0.04, RMSEA = 0.64. Student self-efficacy had a significant and positive direct effect on life satisfaction but not on the perception of access to decent work. Perceived job instability had a significant and positive direct effect on access to decent work but not on life satisfaction. Estimates for pathways are reported in Table 4.

Using multiple-group SEM we tested invariance about the three countries to confirm that the model was adequate for Italian, Swiss, and Spanish students. Analyses confirmed a weak factor invariance (ΔCFI = 0.007, and ΔRMSEA = 0.000) and structural invariance (ΔCFI = 0.001, and ΔRMSEA = 0.000).

## 4. Discussion

In the present study, the aim was to explore the relationships between student self-efficacy, perceived job instability, perception of access to decent work, and life satisfaction in Italian, Spanish and Swiss Higher education students. These results can be useful for the design of guidance and job-training actions for university students, as well as for more enabling behaviors on the companies and policy side in these processes. Numerous studies have shown the importance of studying the perceptions of university students with job-related aspects in relation to career self-management, performance and success in the transition from training to work [61,62].

The results support the idea that perceived job instability correlates negatively with student self-efficacy, decent work access, and life satisfaction. This finding is consistent with Social-Cognitive theory and receives support from other works. Based on their findings, Etehadi and Katarepo [63] suggest that job insecurity erodes self-efficacy, as it hinders employees’ development. Finally, the negative relation between perceived job instability, subjective well-being, and life satisfaction that we have found in this work is consistent with many empirical studies [7,9,10,64].

Job insecurity is one of the most important factors that hinders work [9]. It is a subjective perception [7,26] that involves feelings of uncontrollability and powerlessness which negatively influences the perception of well-being [9]. However, although Spanish students have higher levels of perceived job instability than Italian and Swiss students, they report higher levels of life satisfaction. This perception of job insecurity is consistent with the fact that job insecurity is a structural trait of the Spanish labor market, which is characterized by unemployment and the presence of many temporary and precarious contracts [31]. Among the three countries involved in the study, Spain is the country with the highest youth unemployment rate (June 2022 data: Spain, 28.9%; Italy, 23.7%; and Switzerland, 8.6%). Job insecurity characterizes the transition of Spanish youth into the world of work [65]. Despite this, Spanish students participating in this study show the highest rates of life satisfaction. One explanation may lie in the fact that life satisfaction may differ according to cultural and contextual values. In this sense, the research conducted by Marques et al. [66] found that Spanish students’ life satisfaction is largely explained by social criteria. Family and friendship relationships seem to occupy a preeminent place among the factors influencing the life satisfaction of these young people. A similar conclusion was reached in the report conducted by the Spanish Institute of Youth some years earlier [67].

When considering access to decent work, however, Swiss students have a more positive perception than Italian and Spanish students. Empirical research developed within the framework of the Psychology of Working Theory (PWT; [68]), provides evidence that indicates that the marginalization and economic constraints negatively predict the access to decent work [39]. This could explain why Swiss students, located in a country with better labor market data than in the EU and the OECD, have a better perception of their opportunities of getting decent work. The cited work by Masdonati et al. [39] concludes that contextual and social-psychological factors play an important role in access to decent work and well-being.

Regardless of these differences between Spanish, Italian, and Swiss students, the present study has identified a structural model of relationships that is applicable in all the three contexts. In this model, the perception of access to decent work correlates positively with life satisfaction and it is a partial mediator of the perceived job instability-life satisfaction relationship. Moreover, student self-efficacy directly affects life satisfaction. These findings are consistent partially with the conclusions of Probst et al. [26], who explain that self-efficacy helps to overcome barriers and difficulties in accessing a decent job and, therefore, improves life satisfaction. A high degree of self-efficacy could favor the awareness of responsibility for one’s own destiny and, therefore, improve self-confidence and resilience, increasing the levels of well-being at work and life satisfaction [69]. According to these authors, the inclusion of sustainable development elements (such as decent work) in workplaces would favor feelings of self-efficacy and well-being and, as a consequence, life satisfaction. This would be consistent with our finding on the relationship between access to decent work and life satisfaction. Recent studies in different European countries have analyzed the relationship between access to decent work and life satisfaction [39,40,41,42,43], emphasizing this relationship in accordance with our findings.

According to Singh et al. [69], the inclusion of sustainable practices has a moderating role on the relationships between self-efficacy and job satisfaction. This is consistent with what the present work has shown: student self-efficacy had a significant and positive direct effect on life satisfaction but not on the perception of access to decent work.

As noted above, our findings suggest that student self-efficacy influences life satisfaction positively. The impact of self-efficacy on life satisfaction has been evidenced in numerous studies [70,71]. In a context of job insecurity and uncertainty, the perception of self-efficacy has been shown to be a resilience factor that allows stressors to be moderated and thus promotes life satisfaction. This finding is consistent with the resilience model proposed by Hajek & König [72].

## 5. Conclusions and Limitations

Universities and all other educational institutions need to contribute to the well-being of their students and the development of personal resources that can enable them to make sense of transitions and imagine a positive professional future. This can have an impact on students’ motivation to strive to improve their professional status. Indeed, people are able to project themselves into future scenarios and on the basis of this projection they derive the motivation to adjust their behavior [18]. Imagining a positive professional future could induce university students to take action to make this possible. A threat to this is the perception of job instability, which in our century can be determined by lower employability and underemployment [73]. In fact, our study showed that although it has no effect on the life satisfaction of university students, perceived job instability influences the perceived opportunity to have access to a decent job. On the other hand, student self-efficacy plays a positive role in students’ well-being. These perceptions should be considered by counselors in their work with university students to provide them with personal resources to cope successfully with their transition to work. They should also be considered by the companies with which students’ internships are planned and which receive recent graduates as employees.

Our study is not without some limitations. Firstly, it is a cross-sectional study in which self-report measures were used to collect data. This could produce biased effects. The sampling method used, convenience sampling, could make it difficult to generalize the results obtained. In fact, one of the limitations is the imbalance of the sample in relation to the sex variable. Future studies will work towards a more representative and balanced sample. However, it is worth noting that in this type of university study, the proportion of women is potentially higher. The use of a single item as an indicator of the perceived opportunity to have access to decent work could be reductive, so in subsequent research validated and more reliable tools could be used. Finally, in the future, researchers should study the effect of other variables, including personal ones, affecting the construction of a positive idea of one’s professional future. Furthermore, they should combine data on students’ perceptions of the labor market and objective data on the labor market.

## Figures and Tables

**Table 1:** Characteristics of the sample.

Nationality	N (%)	Gender (Male/Female)	Age (M, SD)
Italian	181 (35)	41/132	22.43 (2.67)
Swiss	173 (33.5)	35/146	22.52 (2.37)
Spanish	163 (31.5)	28/135	22.55 (2.81)
Total	517	104/413	22.50 (2.61)

Note. N = number of participants: M = average; DS = standard deviation.

**Table 2:** Differences between the groups with regard to the dimensions investigated.

	Switzerland (n = 173)		Italy (n = 181)		Spain (n = 163)		Anova	
	M	DS	M	DS	M	DS	*p*	Difference
Perceived Job Instability ^a^	2.41	0.84	2.46	0.94	2.83	0.93	n.s.	Switzerland-Italy
							**0.000**	**Switzerland-Spain**
							**0.001**	**Italy-Spain**
Student self-efficacy ^a^	4.65	0.84	4.72	0.89	4.69	0.77	n.s.	Switzerland-Italy
							n.s.	Switzerland-Spain
							n.s.	Italy-Spain
Decent work access ^b^	3.28	1.15	2.36	0.97	2.26	0.88	**0.000**	**Switzerland-Italy**
							**0.000**	**Switzerland-Spain**
							n.s.	Italy-Spain
Life satisfaction ^b^	4.67	1.26	4.41	1.26	5.28	1.08	n.s.	Switzerland-Italy
							**0.000**	**Switzerland-Spain**
							**0.000**	**Italy-Spain**

Note. ^a^ The hypothesis of homoschedasticity of variances was fulfilled. A one-way ANOVA with Bonferoni’s post hoc test was conducted. ^b^ The assumption of homoschedasticity of variances was not met. A robust Welch’s test with Games–Howell post hoc was conducted.

**Table 3:** Bivariate correlations between model variables and age.

	1	2	3	4	5
Age	-				
2. Perceived Job Instability	−0.07	-			
3. Student self-efficacy	0.10 *	−0.24 **	-		
4. Access to decent work	−0.05	−0.61 **	0.02	-	
5. Life Satisfaction	−0.08	−0.15 **	0.26 **	0.14 **	-

Note: * *p* < 0.05, ** *p* < 0.01.

**Table 4:** Results of SEM analysis.

Link			B	SE	β	*p*
Perceived job instability	→	Access to decent work	−0.28	0.07	−0.20	**
Perceived job instability	→	Life satisfaction	−0.12	0.07	−0.09	n.s.
Student self-efficacy	→	Access to decent work	−0.05	0.09	−0.03	n.s.
Student self-efficacy	→	Life satisfaction	0.46	0.08	0.25	**
Decent work access	→	Life Satisfaction	0.11	0.04	0.12	*

Note. B = unstandardized beta; SE = standard error; β = standardized beta; *p* = significance level. * *p* < 0.05, ** *p* < 0.001.

## Data Availability

The data are available from the corresponding author upon reasonable request.
